# Multiple Phenotypes in Adult Mice following Inactivation of the Coxsackievirus and Adenovirus Receptor (*Car*) Gene

**DOI:** 10.1371/journal.pone.0020203

**Published:** 2011-06-03

**Authors:** Ahmad Pazirandeh, Taranum Sultana, Momina Mirza, Björn Rozell, Kjell Hultenby, Karin Wallis, Björn Vennström, Ben Davis, Anders Arner, Rainer Heuchel, Matthias Löhr, Lennart Philipson, Kerstin Sollerbrant

**Affiliations:** 1 Ludwig Institutet for Cancer Research, Stockholm Branch, Stockholm, Sweden; 2 Department of Women's and Children's Health, Karolinska Institutet and University Hospital, Stockholm, Sweden; 3 Department of Laboratory Medicine, Karolinska Institutet and University Hospital, Huddinge, Sweden; 4 Department of Cell and Molecular Biology, Karolinska Institutet, Stockholm, Sweden; 5 Department of Physiology and Pharmacology, Karolinska Institutet, Stockholm, Sweden; 6 Department of Clinical Science, Intervention and Technology, Karolinska Institutet, Huddinge, Sweden; French National Centre for Scientific Research, France

## Abstract

To determine the normal function of the Coxsackievirus and Adenovirus Receptor (CAR), a protein found in tight junctions and other intercellular complexes, we constructed a mouse line in which the CAR gene could be disrupted at any chosen time point in a broad spectrum of cell types and tissues. All knockouts examined displayed a dilated intestinal tract and atrophy of the exocrine pancreas with appearance of tubular complexes characteristic of acinar-to-ductal metaplasia. The mice also exhibited a complete atrio-ventricular block and abnormal thymopoiesis. These results demonstrate that CAR exerts important functions in the physiology of several organs *in vivo*.

## Introduction

Coxsackievirus and adenovirus receptor (CXADR or CAR) is a cell adhesion molecule that acts as a cellular receptor for both coxsackie B and many species of adenovirus, and has attracted attention for its importance in virus-related disease and for the development of adenovirus as a vector for gene therapy [1].

CAR belongs to a subfamily within the immunoglobulin superfamily. It contains a membrane-distal V-type and a membrane-proximal C2-type immunoglobulin domain, a single transmembrane region, and a cytoplasmic tail. Members of this subfamily including junctional adhesion molecules (JAMs), endothelial-cell-selective adhesion molecule (ESAM) and CAR are mainly expressed on the surface of endothelial and epithelial cells where they regulate the migration of immune cells across endothelial and/or epithelial barriers and aid in the establishment of cell polarity and barrier function of endothelial and/or epithelial tissues [2], [3].

Expression of the *Car* gene is developmentally regulated. Mouse embryos express the protein abundantly, whereas expression in the adult mouse is more restricted and confined mainly to the tight junctions of epithelial cells where CAR contributes to barrier function [1], [4], [5]. The CAR protein is also localized to neuromuscular junctions, intercalated discs in the heart and to the acrosome region of germ cells in the testis [6], [7].

A functional role of CAR in cardiac development has recently been demonstrated. Targeted disruption of CAR is embryonically lethal due to heart failure early in embryogenesis [8], [9], [10]. Importantly, embryos with cardiac-specific deletion of CAR induced after embryonic day 11 (E11) are viable but develop an atrioventricular heart block (AV-block) that is maintained to adulthood without any increase in mortality [10], [11]. A complete AV-block also develops in adult mice with a cardiac-specific depletion of CAR. In these mice an altered localization of connexin 45, ß-catenin and zonula occludens-1 (ZO-1) preceded development of cardiac dysfunction [11]. Moreover, a defective communication through tight- and gap junctions in cardiomyocytes has been suggested [12]. In contrast, cardiac-specific overexpression of CAR causes disorganization and degeneration of cardiomyocytes, disrupted adherens junctions, cardiomyopathy and ultimately animal death [13]. Overexpression of CAR driven by a skeletal muscle-specific promoter results in a severe and lethal myopathic phenotype [14]. These results indicate that a tight regulation of CAR protein levels is required for proper muscle tissue function. Ubiquitous over-expression of the extracellular and transmembrane domains of CAR does not result in any obvious animal phenotype *in vivo*, showing that the complete protein including the intracellular tail region is required for the downstream signalling and the physiological effects of CAR [14].

The signalling pathways that modulate CAR-mediated functions are not well understood. CAR protein levels are dynamically regulated by inflammatory responses and signals that control proliferation and differentiation including the Raf/MEK/ERK, FSH and TGF-ß mediated signalling pathways [15], [16], [17], [18], [19], [20], [21], [22]. CAR has also been associated with an activation of both β-catenin- MAPK and PI3K signaling pathways [13], [23], [24].

Several PDZ-domain containing proteins including ligand-of-numb protein-X (LNX), ligand-of-numb protein-X2 (LNX2), zonula occludens-1 (ZO-1), membrane-associated guanylate kinase 1b (MAGI-1b), protein interacting with protein C kinase (PICK1), postsynaptic density95 (PSD-95) and multi-PDZ domain protein-1 (MUPP1) interact with the intracellular tail of CAR suggesting that CAR is present in larger protein complexes [5], [25], [26], [27], [28]. In addition, CAR can form homophilic interactions with CAR molecules on neighbouring cells as well as with JAM-C and JAML suggesting that cross talk occurs between members of this immunoglobulin subfamily [7], [29], [30]. CAR is also found in the same protein complex as connexin 45, a gap junction protein, and with components of the cytoskeleton [11], [31], [32].

Since deletion of the *Car* gene produces an embryonic lethal phenotype it has not been possible to analyse the integrated function of CAR in a broader range of tissues in the adult organism. We have therefore developed a mouse strain with a conditional ablation of *Car* in order to carry out temporally controlled global inactivation of the *Car* gene. Here we demonstrate a role for CAR in the physiology of the heart, pancreas, intestine and thymus in adult mice.

## Methods

### Ethics Statement

All animal experimentation was conducted in accordance with accepted standards of humane animal care, and was approved by Stockholm North Animal Ethical Board (N179/08).

### Generation of conditional *Car* knockout mice

Mice with a loxP-flanked *Car* allele (F/F mice) were generated at the MCI/ICS (Mouse Clinical Institute - Institut Clinique de la Souris-, Illkirch, France; http://www-mci.u-strasbg.fr). Three fragments corresponding to a 4.3 kb 5′ homology arm, a 0.4 kb fragment harbouring exon-2 and two flanking loxP sites, and a 2.8 kb 3′ homology arm were amplified by PCR from 129S2/SvPas ES cells and subcloned in an MCI proprietary vector harbouring a neomycin selection cassette flanked by flippase recognition target sites. The linearized construct was electroporated into 129S2/SvPas mouse embryonic stem (ES) cells. After selection, targeted clones were identified by PCR using external primers and further confirmed by Southern blot with 5′ and 3′ external probes. The neomycin cassette was removed and two positive ES clones were injected into C57Bl/6J blastocysts, and male chimaeras generating germline transmission were identified and used for further breeding.

F/F mice were then crossed with the transgenic mouse line B6.Cg-Tg (CreEsr1)5AmC/J (purchased from The Jackson Laboratory) expressing a tamoxifen-inducible Cre-ERTM fusion protein under the control of a chicken ß actin/cytomegalovirus (CMV) promoter [33]. Additional breeding created the mouse line F/F;Cre that was backcrossed three times onto C57Bl/6J and then used for experiments.

Cre-mediated deletion of exon 2 results in a frame shift and premature termination within the CAR leader sequence, creating a null allele. The first exon comprising 15 of the 19 amino acids that constitute the signal peptide remain intact. Following the frameshift, the transcript from the null allele encodes 9 amino acids unrelated to CAR (HFVFWRQNL) followed by a stop codon. No abnormal transcripts were observed in tissues from cKO animals when analyzed by RT-PCR using primers for the 5′ and 3′ ends of the transcript (data not shown), and Western blot analyses using all our in house CAR antibodies (IG1, RP1284 and RP291) did not reveal any shortened CAR variants.

### Genotyping

Genotyping of mice was performed by PCR amplification of genomic DNA isolated from the tail. Primer pairs (GAGACTGGATTATGAGTTCCAGGCTTTAG) and (GCTATTCATGGGAGCAAAGTGATACAGG) were used to amplify an area encompassing one of the loxP sites. The size of the DNA fragments was 211 bp for the wildtype (wt) allele and 261 bp for the floxed allele. Cre-specific primers (GCGGTCTGGCAGTAAAAACTATC) and (GTGAAACAGCATTGCTGTCACTT) amplified a 100 bp fragment in animals harbouring the Cre transgene. Following tamoxifen treatment, the *Car* null allele was identified with primers (GAGACTGGATTATGAGTTCCAGGCTTTAG) recognizing *Car* on chr 16 position 74433415–74433443 (NW001030584) and (CCTGCTCCAGATTCCCACAATTCC) recognizing *Car* on chr 16 position 74434106–74434129 (NW001030584.1) that amplified a region encompassing both loxP sites and the second exon. The size of the amplified DNA fragment was reduced from 874 bp (floxed allele) to 409 bp (*Car* null allele). The size of the corresponding wt allele was 741 bp.

### Nomenclature

The following nomenclature is used for mice throughout the paper. +/+ animals are wt C57Bl/6J mice, cKO are tamoxifen-treated animals with the genotype F/F;Cre, Ctrl are control animals with genotype +/+ or F/F as indicated in the text, +/+;Cre animals express the Cre recombinase but do not contain any loxP sites.

CAR and *Car* is used throughout the paper and refers to the protein and the CXADR gene, respectively.

### Tamoxifen treatments

Tamoxifen (Sigma, T5648) was dissolved in corn oil (Sigma, C8267) to a final concentration of 10–20 mg/ml by vigorous shaking at 55°C for a couple of hours. Dissolved tamoxifen was prepared fresh and used within a week. Adult animals (minimal age 3 weeks) were injected intraperitoneally with tamoxifen (0.1 mg/g body weight) once a day for five consecutive days (day 1–5). If not stated otherwise animals were given a booster dose one week later (day 12). Mice were generally sacrificed on day 19 (standard protocol). In some cases additional booster doses were given, and the day for sacrifice was prolonged (up to 18 months) as indicated in the text.

### Tissue preparations and analyses

For histology, the gastrointestinal tract was removed from the abdominal part of the oesophagus to the rectum. The mesentery containing the pancreas as well as regional lymph nodes was bluntly removed from the intestine apart from the most proximal part. The intestine was immersed in phosphate buffered 4% formalin (Solveco AB, cat # 1198), fixed for 24 hrs and then kept in 70% ethanol until analysis. The proximal intestine with attached pancreas and mesentery was laid flat on a piece of paper and fixed for a total of 24 hours, dehydrated and embedded in paraffin according to standard procedures. Paraffin sections were dewaxed and stained with haematoxylin/eosin.

For Transmission Electron Microscopy (TEM) tissue was dissected and small pieces were fixed in 2% glutaraldehyde +0.5% paraformaldehyde in 0.1 M sodiumcacodylate buffer containing 0.1 M sucrose and 3 mM CaCl_2_, pH 7.4 at room temperature for 30 min followed by 24 hours at 4°C. Specimens were rinsed in 0.15 M sodiumcacodylate buffer containing 3 mM CaCl_2_, pH 7.4 postfixed in 2% osmium tetroxide in 0.07 M sodiumcacodylate buffer containing 1.5 mM CaCl_2_, pH 7.4 at 4°C for 2 hrs, dehydrated in ethanol followed by acetone and embedded in LX-112 (Ladd, Burlington, Vermont, USA). Semithin sections were cut and stained with Toluidinblue O (Merck, Darmstadt, Germany) and used for light microscopic analysis. Ultrathin section (approximately 40–50 nm) were cut and contrasted with uranyl acetate (Merck, Darmstadt, Germany) followed by lead citrate (Merck, Darmstadt, Germany) and examined in a Leo 906 transmission electron microscope at 80 kV (Park *et al*, 2007). Digital images were taken by using a Morada digital camera (Soft Imaging System, GmbH, Münster, Germany).

For analyses of thymocytes and T-cell sub populations the thymus and spleen were passaged through a steel net. Cells were washed and resuspended in cold, complete RPMI medium (Gibco, 61870) supplemented with 50 IU/ml penicillin, 50 mg/ml streptomycin, 2 mM L-glutamine and 10% heat-inactivated fetal calf serum, all obtained from Gibco-BRL (Paisley, Scotland). Splenocytes were treated with red blood cells lysing buffer (Sigma, R7757) for a 5 minutes at room temperature to lyse the red blood cells. Leukocytes were washed twice and resuspended in complete RPMI medium supplemented with 50 IU/ml penicillin, 50 mg/ml streptomycin, 2 mM glutamine and 10% fetal calf serum. Thymocytes and purified leukocytes from spleen and blood were stained with anti-CD4-FITC and anti-CD8-PE antibodies in ice-cold complete RPMI medium for 1 h on ice. After washing, 10,000 cells were analyzed by flow cytometry in a FACSscan (Becton Dickinson and Co., Franklin Lakes, NJ). Cells were gated to exclude debris and clumps.

### Indirect double immunofluorescence, protein extraction and Western blot analysis

These methods have been described previously [7], [22], [25]. Modifications from the described methods were that mounting media containing 4′,6-diamidino-2-phenylindole (DAPI) (VectraSheild H1200 ) was used in the double immunofluorescence analyses and that preparation of protein extracts from certain tissues required both homogenization and sonication in ice-cold urea lysis buffer (8 M urea, 0.2% SDS, 0.8% Triton-X100, 3% 2-mercaptoethanol and complete protease inhibitors) [4]. Protein concentrations in cell extracts were quantified by conventional Bradford analysis or by using a commercially available kit (Bio-Rad Laboratories, 500-0006). The same concentration of SDS and Triton X-100 used in the lysis buffer was also used in the blank and standards. Calnexin was included as a loading control in the Western blot analyses.

### Antibodies

Rabbit, polyclonal anti-CAR antibodies (IG1, RP1284 and RP 291) and the antibody towards Calnexin have been described previously [4], [34]. The antibody towards IG1 recognizes the first immunoglobulin loop in the CAR protein. RP291 recognizes the C-terminal region of CAR-2 (VMIPAQSKDGSIV in the human protein). RP1284 recognizes amino acids 315–331 present in the cytoplasmic tail of mouse CAR common to both major isoforms (KTQYNQVPSEDFERAPQ). All CAR antibodies recognize both mouse and human CAR proteins. The rat monoclonal anti-occludin antibody (MOC37) was a kind gift from Dr. M. Furuse, Kobe University, Japan. The rat monoclonal anti-ZO-1 antibody was purchased from Chemicon (MAB1520). For flow cytometry anti-CD4-FITC and anti-CD8-PE monoclonal antibodies (11-0041-81 and 12-0081-81, EBioSciences San Diego, CA) were used. Secondary antibodies were donkey anti-rabbit Alexa Fluor 488 (A21206, Molecular Probes Inc, Eugene, OR) donkey anti-rat Alexa Fluor 594 (A21209 Molecular Probes Inc, Eugene, OR) and HRP labelled anti-rabbit Ig (NA934V, GE health care, UK).

### Urine analysis

Combur-Test (Roche) strips were used on fresh voided urine for semi quantitative assessment of pH, leukocytes, nitrate, protein, glucose, ketones, urobilinogen, bilirubin, hemoglobin, and red blood cells.

### Blood analysis

Blood samples were collected from the tail into serum separator microtubes containing no anticoagulants, then centrifuged and frozen until analysis. The concentrations of different biochemical parameters were determined in individual serum samples using an autoanalyzer.

### Shirpa test

The Shirpa primary screen was used to assess the behavioural and functional profile on mice 2–6 months of age [35]. A clear Perspex cylinder with a 14 cm diameter and 18 cm height placed on tripod of 10 cm height was used for the evaluation of undisturbed behaviour for 5 min. Subsequently, the mouse was transferred to an arena (54×32 cm) for observation of motor behaviour. Thereafter, a sequence of manipulations using tail suspension and supine restraint was performed to evaluate parameters such as grip strength, limb tone, and reflexes. The procedure was ended by the measurement of body weight. The equipment was cleaned with 70% ethanol between animals. Scoring was performed as proposed by Hatcher et al [36] with some modifications as suggested by the standard operating procedures in Eumorphia EMPReSS (http://empress.har.mrc.ac.uk/). Respiration, heart rate, salivation, fear, irritability, and body temperature were not assessed.

### Electrocardiography (ECG)

28–37 week old mice were anesthetised with isoflurane (Forene, Baxter, USA). Needle electrodes were inserted under the skin in the thoracic region rostral and caudal to the heart. ECG signals were captured using BioAmp (AD Instruments, Australia) and Powerlab (ADInstruments Ltd, Oxfordshire, UK) systems, and analysed using Chart v4.2 (ADInstruments). For each animal at least 30 seconds of data was recorded and analysed for rhythm. Representative regions with minimal movement artefacts were used to analyse atrial and ventricular rates and PQ intervals.

### Assessment of intestinal permeability

Intestinal permeability was assessed as described in [37] using fluorescein-isothiocyanate-conjugated dextran-4000 (FITC-dextran) as a permeability probe. FITC-dextran (Sigma Chemical Co.) was prepared in a concentration of 20 mg/ml in PBS. 30 mg/100 g body weight was administered by gavage 4 h prior to sacrifice.

### Statistical methods

Data are presented as mean ± SD or mean ± SEM, as stated in the text. For data analysis, a two-tailed Student's *t* test was used and *p*<0.05 was considered significant. The number of experiments is indicated in the text.

## Results

### Generation of conditional *Car* knockout mice

**Figure 1:**
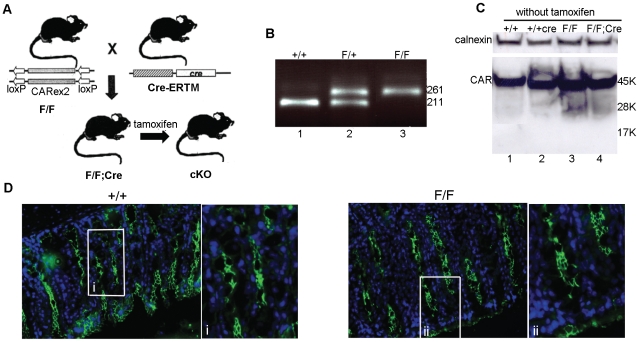
Construction of a conditional *Car* knockout mouse. (*A*) Schematic presentation of the targeting strategy. Tamoxifen is required to produce a *Car* null allele (cKO). (*B*) PCR analysis of genomic DNA isolated from the mouse tail. The genotype of wt (+/+), heterozygote (F/+) and homozygote (F/F) mice is indicated. The 261 bp and 211 bp PCR products corresponds to floxed and wt alleles respectively. (*C*) Western blotting of protein extracts prepared from pancreas shows that insertion of loxP sites and the Cre transgene do not affect CAR protein levels in the absence of tamoxifen. Genotypes and molecular weight markers are indicated. The CAR-specific antibody RP291 was used to detect CAR. The same result was obtained with the antibody RP1284 (data not shown). Calnexin was used as a loading control. The experiment was performed >3 times. (*D*) Indirect immunofluorescence on sections from large intestine was used to demonstrate that insertion of loxP sites did not affect the subcellular localization of the CAR protein in the absence of tamoxifen. Genotypes are indicated. The CAR-specific antibody RP291 was used to detect CAR (green). The same result was obtained when the IG1 antibody was used to detect CAR (data not shown). DAPI was used to visualize nuclei (blue). Magnification was 40×. (i) and (ii) are enlargements of the boxed areas in the +/+ and F/F image, respectively. The experiment was performed 3 times.

We constructed a floxed *Car* allele that can be disrupted at any chosen time point by tamoxifen-regulated Cre-mediated recombination *in vivo* (Fig. 1A). Genotypes were determined by PCR amplification of genomic DNA isolated from the tail (Fig. 1B and data not shown).

RT-PCR, Western blot and indirect immunofluorescence analyses did not reveal any difference between wt (+/+) and floxed (F/F) mice regarding CAR mRNA expression, total amount of CAR protein or subcellular localization of CAR in any organ tested (Figs. 1C and D, and data not shown). We therefore conclude that insertion of loxP sites in the non-coding parts surrounding the 2^nd^ exon does not affect expression from the *Car* gene. Similarly, introduction of the Cre expression cassette did not affect CAR expression in the absence of tamoxifen, since Western blot and indirect immunofluorescence analyses showed that CAR protein levels and subcellular localization were indistinguishable between wt (+/+) and mice harbouring the Cre transgene (Figs. 1C and data not shown). In addition, indirect immunofluorescence using a Cre-specific antibody confirmed that no nuclear Cre protein was found in organs isolated from F/F;Cre animals, and no DNA excision originating from recombination between the two loxP sites was detected in PCR analysis (data not shown). We conclude that the Cre protein does not cause any detectable recombination in F/F;Cre mice in the absence of tamoxifen and that neither the F/F nor the F/F;Cre mice in the absence of tamoxifen differ from wildtype mice with regard to CAR gene expression.

### Tamoxifen induce an efficient Cre-mediated inactivation of the *Car* gene

To determine whether administration of tamoxifen could induce recombination between the loxP sites, littermates of F/F;Cre (cKO) and F/F controls (Ctrl) were given tamoxifen by intraperitoneal injections. Mice were sacrificed between one week and 18 months after the last tamoxifen administration and analyzed by PCR and Western blotting.

**Figure 2:**
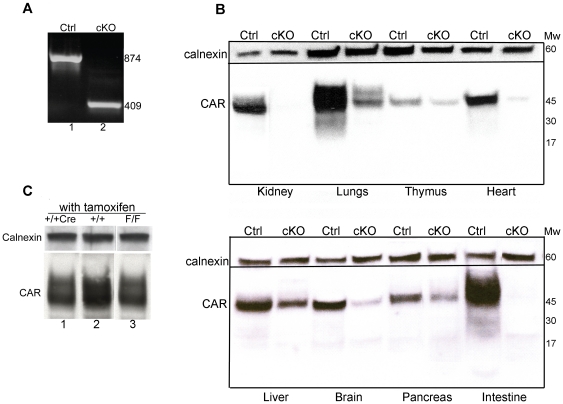
Inactivation of the *Car* gene following tamoxifen administration. (*A*) Genomic tail DNA was isolated from tamoxifen-treated animals with genotypes F/F (ctrl, lane 1) and F/F;Cre (cKO, lane 2). PCR amplification demonstrated that an efficient excision of exon 2 was specifically obtained in the cKO animals. (*B*) Western blot analysis of organ extracts from tamoxifen-treated F/F control (Ctrl) and F/F;Cre (cKO) animals. The CAR-specific antibody RP291 was used to detect CAR. The same result was obtained with RP1284 (data not shown). Calnexin was used as a loading control. The experiment was performed at least three times. (*C*) Western blotting of protein-extracts prepared from lungs of tamoxifen-treated mice. No loss of CAR protein is found in animals with genotypes +/+;Cre (lane 1) or F/F (lane 3) as compared to wt controls (lane 2) in the presence of tamoxifen. The CAR-specific antibody RP291 was used to detect CAR. The same result was obtained with RP1284 (data not shown). Calnexin was used as a loading control. The experiment was performed at least three times.

PCR performed on DNA isolated from the tail demonstrated an efficient excision of the second exon specifically in the cKOs (Fig. 2A). The same result was seen when PCR was performed on DNA isolated from other organs including kidney, heart and spleen (data not shown). One notable exception was the liver, in which the recombination was much less efficient compared to the other organs tested (data not shown). This observation was in accordance with a previous report, demonstrating reduced efficiency of the CMV-Cre-ERTM system in the liver [33]. The low recombination efficiency in the liver might reflect a metabolization and subsequent inactivation of the drug.

Protein extracts were prepared from different organs and the expression of CAR protein was analyzed by Western blotting. Inactivation of the *Car* gene in cKO animals was associated with a significant decrease in CAR protein levels in most organs analyzed (Fig. 2B). The amount of CAR protein in the liver was still relatively high in the cKO, which correlated well with the results of the PCR analysis. The low CAR protein levels were maintained for at least 18 months after the last tamoxifen administration (length of experiment). These results show that the *Car* gene can be irreversibly inactivated in a broad range of tissues in adult F/F;Cre mice in the presence of tamoxifen.

The CAR protein levels in tamoxifen-treated F/F and +/+;Cre control animals were indistinguishable from wt demonstrating that neither tamoxifen treatment itself nor the presence of the Cre protein alone can disrupt expression from the *Car* gene (Fig. 2C).

### Metabolism, fertility, behaviour and locomotion activity in cKO mice

**Figure 3:**
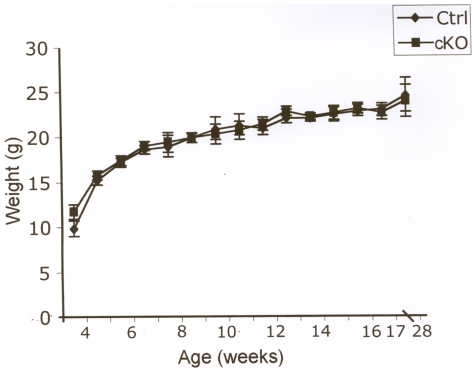
Body weight of cKO and littermate controls after tamoxifen administration. The body weights of F/F;Cre (cKO, n = 20) and F/F littermate control (Ctrl, n = 21) animals were measured weakly following tamoxifen administration given at 4 weeks of age. No difference between the two groups was found. Data are presented as mean ± SEM.

To evaluate metabolism, fertility, behaviour and locomotion activity, tamoxifen was injected into littermate F/F;Cre (cKO) and F/F control (Ctrl) animals. Animals (cKO, n = 20; controls, n = 21) were weighed weekly for six months, however no difference in weight gain between the two groups was observed (Fig. 3). The cKO animals were fertile and appeared healthy with no increase in mortality compared to controls. The cKO animals showed a tendency for early onset of grey fur, but statistical analysis could not confirm the significance of this finding.

Faeces and fresh voided urine samples were obtained from tamoxifen-injected cKO mice (n = 20) and littermate controls (n = 25) over a period of 6 months and colour, pH, leukocytes, nitrate, protein, glucose, ketones, urobilinogen, bilirubin, hemoglobin and red blood cells were analyzed. No differences between the two groups were found (data not shown). Faeces appeared normal in consistency and pellet-size. No sign of constipation, diarrhoea or bleeding was observed.

Serum samples from cKO (n = 17) and littermate controls (n = 18) were collected and the concentration of different biochemical parameters was determined. No significant difference was found between the two groups. The concentration of the serum parameters in cKO vs. controls was as follow. Total Protein (g/L) 52.12±4.34 vs. 51.39±2.59; Albumin (g/L) 33.71±1.65 vs. 33.06±1.06; Amylase (µkat/L) 35.95±7.48 vs. 38.37±6.79; Bile Acid (µmol/L) 7.06±5.37 vs. 9.34±6.51; Cholesterol (mmol/L) 1.68±0.38 vs. 1.84±0.38; Triglyceride (mmol/L) 0.87±0.23 vs. 0.75±0.21; ASAT (µkat/L) 1.12±0.43 vs. 0.9±0.25; Creatinin (µmol/L) 30.94±17.36 vs. 26.53±12.4.

An observational assessment of locomotor function and sensory system behavior using the SHIRPA primary screen of standardized protocols was done on tamoxifen-injected F/F;Cre (cKO, n = 13) and F/F littermate controls (Ctrl, n = 10). Only one of the 35 tests showed a significant difference between cKO and control mice, the transfer arousal test, which measures the immediate reaction to a new environment. The cKO mice showed a lower tendency to freeze and instead moved immediately. In this test 46% of the cKO mice froze momentarily before starting to explore and 54% immediately explored the area, as compared to the control mice of which 80% froze momentarily and only 10% showed immediate movement (data not shown). Extended freezing would imply anxiety or fear. The behavior of the control mice, to freeze for not more than 5 seconds initially, is however considered normal. If the lack of freezing of the cKO mice would be caused by hyperactivity, that behavior was not recorded in the viewing jar or in locomotor activity and the importance of this finding is therefore unclear.

### Dilated intestines and atrophy of the exocrine pancreas in cKO mice

**Figure 4:**
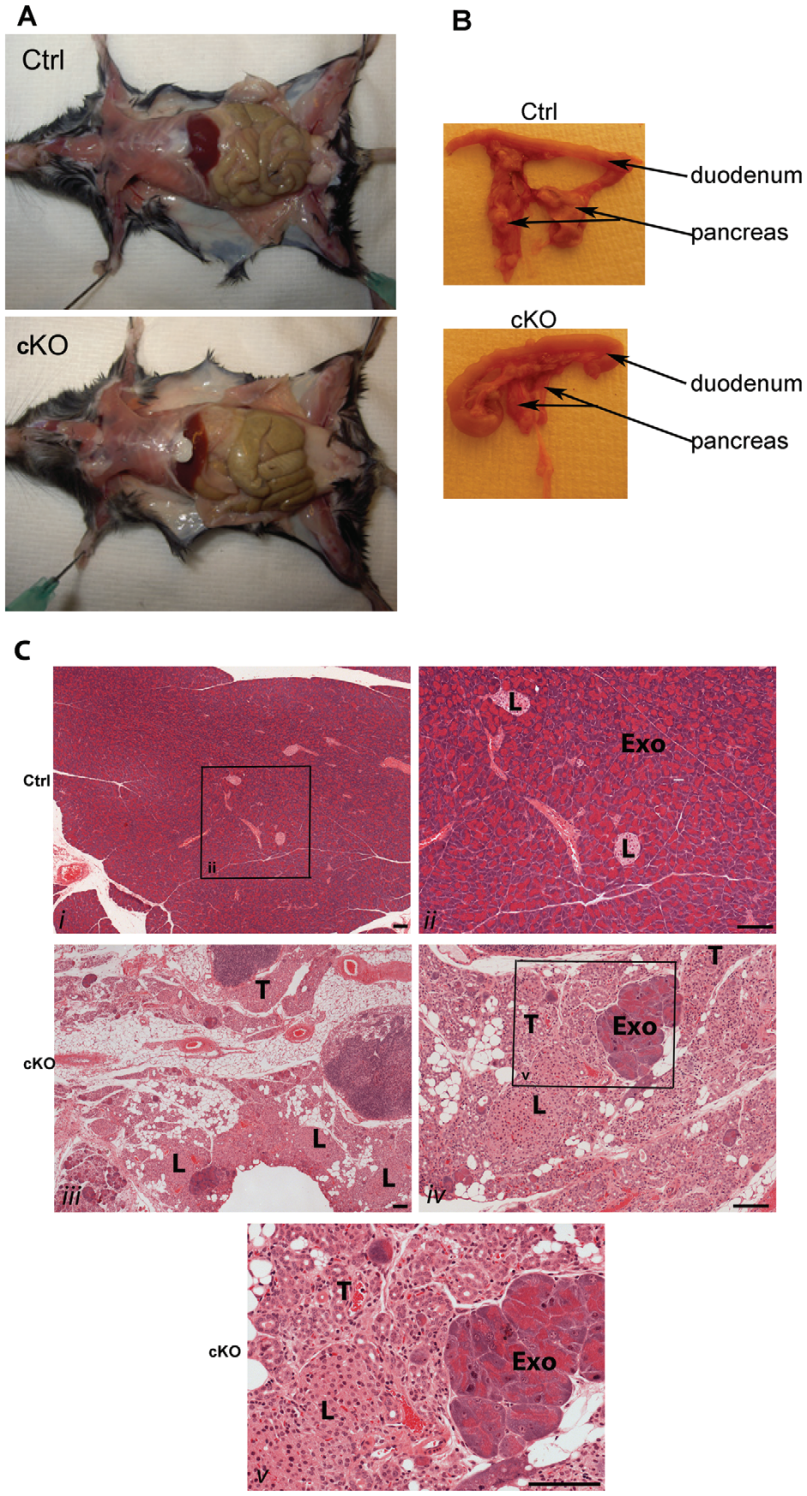
Enlarged intestines and atrophy of exocrine pancreas in cKO mice. (*A*, *B*) Necropsy of tamoxifen-treated F/F;Cre (cKO) and F/F controls (Ctrl) revealed enlarged intestines (*A*) and a dramatic reduction in the size of pancreas (*B*) in cKO animals. Analysis was repeated >30 times. (*C*). Histology of pancreata tissue harvested two weeks after the last tamoxifen administration from F/F control (Ctrl) and F/F;Cre (cKO) animals. (i, ii) Control animals displayed an organized structure of exocrine pancreas (Exo) with interspersed Islets of Langerhans (L). Acinar cells were polarized with more basophilic staining (blue) corresponding to endoplasmic reticulum and accumulation of secretory granules at the apical side. (ii) is a magnification of the boxed area indicated in (i). (iii–v) Pancreas of cKO mice displayed atrophy of exocrine pancreas, enrichment of adipose tissue and apparently normal Islets of Langerhans (L). Only small parts of normal exocrine pancreas could be found (Exo). The acinar cells were replaced by duct-like tubular complexes (T). (v) is a magnification of the boxed area indicated in (iv). Scale bar = 100 µm.

Littermates of F/F;Cre (cKO, n>30) and F/F controls (Ctrl, n>30) were treated with tamoxifen. Necropsy one week after the last tamoxifen administration revealed major macroscopic changes in the intestinal system and pancreas (Figs. 4A and B). The intestine of all cKO animals analyzed was dilated (Fig. 4A). The overall length of the gastrointestinal tract was not affected (data not shown). In addition, the pancreas was found to be dramatically smaller in all cKO mice compared to the controls (Fig. 4B). Interestingly, cKO mice analyzed up to 18 months after the last tamoxifen injection maintained the dilated intestines and the pancreatic atrophy indicating that these effects were irreversible. No polyps or tumors were found.

Tissues were collected from F/F;Cre (cKO, n = 12) and F/F controls (Ctrl, n = 12) animals one week up to 18 months after the last administration of tamoxifen, and analyzed by haematoxylin and eosin staining. Major histological changes were found in the pancreas. While the exocrine pancreas in control mice had large, polarized acinar cells and associated ducts surrounding richly vascularised Islets of Langerhans, the cKO mice demonstrated a striking atrophy of exocrine tissue (Fig. 4C). Pancreas of cKO mice consisted mainly of adipose tissue in which apparently normal Islets of Langerhans were interspersed. There was an almost complete lack of acinar cells and only small parts of apparently normal exocrine pancreas could be found. Duct-like tubular complexes replaced the acinar cells, and the pancreas therefore displayed a metaplastic phenotype. The atrophy and metaplastic phenotype was already present three weeks after the initiation of tamoxifen treatments and was then maintained for more than 18 months indicating that the effect was irreversible. No sign of fibrosis was observed. No obstruction or damage was found in the pancreatic ducts or in the associated ducts in the liver and gallbladder in cKO animals (data not shown).

Neither the dilated intestines nor the pancreatic atrophy were seen in similarly treated +/+;Cre control mice demonstrating that the effects were not caused by expression of the Cre protein alone (data not shown).

An increase in inflammatory cells was observed in the pancreas of both cKO and controls the first weeks after tamoxifen administration. This effect was however not maintained and no sign of pancreatitis were observed in older animals (data not shown).

No other macroscopic or histological changes were found in the cKO and all other organs including intestines, heart and thymus appeared normal. No hemorrhage or edema was found in cKO animals, and both blood- and lymphatic vessels appeared normal based on histology and immunofluorescence staining using endothelial cell markers (data not shown).

### Atrioventricular block in cKO mice

**Figure 5:**
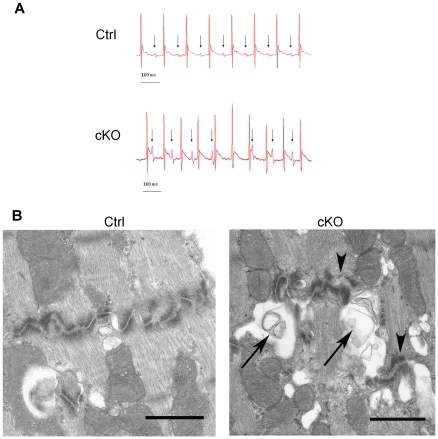
Atrioventricular heart block and ultrastructural changes in hearts of cKO mice. (*A*) Representative ECG recordings of tamoxifen-treated F/F control (Ctrl, upper panel) and F/F;Cre (cKO, lower panel) animals. A complete AV block was demonstrated in the cKO animals while control mice showed normal sinus rhythm. Arrows mark the individual P waves. Note the absence of a constant coupling interval between the P waves and the QRS complexes in cKO mice. (*B*) Transmission electron microscopy of cardiac muscle from tamoxifen-treated F/F control (Ctrl) and F/F;Cre (cKO) animals. A disconnection between myofilaments and big vacuoles containing parts of membranes (arrows) and wider zonula adherens containing a clot like contents (arrow heads) was observed in cKO animals. The experiment was performed on three different animals from each group. Scale bar = 1 µm.

ECG was performed 24 weeks (cKO, n = 10; littermate controls, n = 5) after the last tamoxifen administration. In each animal, at least 30 seconds of ECG recording was examined. All control mice exhibited sinus rhythm, without ectopic beats, and with a mean heart rate of 549±25 min^−1^ (mean ± SD, n = 5, range = 478–589), and a mean PQ interval (measured from the start of the P wave to the beginning of the Q wave) of 38±5 (mean ± SD, n = 5, range 32–49 ms) milliseconds. All examined cKO mice showed a complete atrioventricular (AV) block with temporal dissociation between atrial depolarisation (P waves) and ventricular depolarisation (QRS complexes). Figure 5A shows representative tracings from a control mouse (top panel) and a cKO mouse (bottom panel). The cKO mice showed an average P-wave frequency of 479±133 min^−1^ (mean ± SD, n = 10, range = 248–644), and an average R-wave frequency of 580±47 min^−1^ (mean±SD, n = 10, range = 527–656). The P-wave frequency was significantly slower than the R-wave frequency (P<0.05; Student's t-test). The ventricular frequency in cKO mice was slightly faster than that of their control counterparts, however this effect was not statistically significant (p = 0.20, unpaired Student's t-test). No major difference in the duration of the QRS complexes was observed between the control and the cKO groups.

CAR is expressed in intercalated discs between myocytes [6]. To analyze the junctions between myocytes at the ultrastructural level, transmission electron microscopy was performed on heart tissue from tamoxifen-treated F/F;Cre (cKO, n = 3) and F/F control mice (Ctrl, n = 3). The intercalated discs of the cardiac muscle in control animals displayed a well-defined and characteristic step-like morphology i.e. a tight connection between the myofilaments and abundance of dense material at the intersection (Fig. 5B). In contrast, the intercalated discs of cardiac muscle in cKO animals showed a heterogeneous morphology. Although some areas showed normal morphology, most discs were severely changed. The most striking observation was a disconnection between myofilaments and the presence of big vacuoles-containing parts of membranes. Furthermore the outline of the junction was also changed i.e. in some areas the distance between zonula adherens was wider and contained a clot like contents (Fig. 5B).

ECG recording and transmission electron microscopy performed on similarly treated +/+;Cre control mice were indistinguishable from wt mice demonstrating that the effects seen in cKO animals was not caused by expression of the Cre protein alone (data not shown).

From these experiments we conclude that inactivation of the CAR gene in adult mice causes irreversible AV-block and structural changes in adhesion between myocytes confirming the importance of CAR for normal heart function.

### The total number of thymocytes is increased in cKO mice

**Figure 6:**
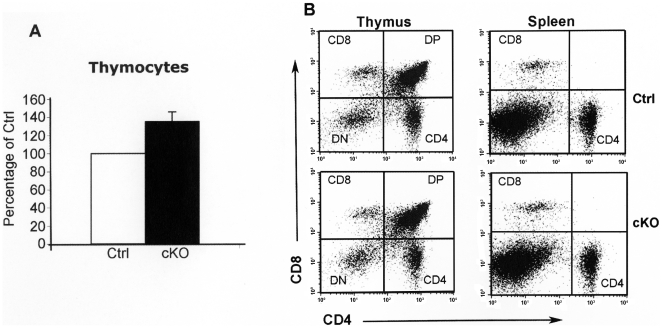
The total number of thymocytes is increased in cKO mice. Total number of thymocytes in tamoxifen-treated F/F;Cre (cKO, n = 6) and littermate F/F controls (Ctrl, n = 6). The number of cells in cKO mice is shown as percent of cells in controls. Data are presented as mean ± SEM. *P*<0.05. (*B*) Flow cytometric analysis of thymocyte populations (thymus) and T cell subsets (spleen) in cKO and control (Ctrl) mice. Single cells are displayed on a dot plot of CD4 versus CD8. CD8: (CD8^+^ single positive); CD4: (CD4^+^ single positive); DP: (CD4^+^/CD8^+^ double positive); DN: (CD4^−^/CD8^−^ double negative). Experiments were performed three times.

To investigate possible effects of CAR on thymocytes and leukocytes, littermates of F/F;Cre (cKO, n = 10) and F/F controls (Ctrl, n = 10) were treated with tamoxifen. When the thymus and spleen were removed 2–3 days after the last tamoxifen administration, thymus was found to be notably larger in cKO than in control animals (data not shown). Thymocytes and spleen leukocytes were isolated, counted, and analysed by flow cytometry. The results demonstrated an increase in the total number of thymocytes in cKO compared to control mice (*P*<0.005) (Fig. 6A). This effect was not seen in +/+;Cre control mice demonstrating that Cre protein alone could not affect the number of thymocytes (98±5.7% of controls). The percentage of cells in the four thymocyte populations was unchanged, indicating that all populations were similarly affected (Fig. 6B). The percentage of different thymocyte populations in cKO vs. controls was as follows. CD8^+^ single positive: 6.08±1.8 in cKO mice vs. 5.15±1.8 in control mice, CD4^+^ single positive: 9.63±1.8 vs. 9.70±2.2, CD4^+^/CD8^+^ double positive (DP): 73.13±6 vs. 74.85±5.4, CD4^−^/CD8^−^ double negative (DN): 10.42±3.7 vs. 9.33±1.5. The increased number of thymocytes in cKO mice was not caused by an increase in cell proliferation since flow cytometric analysis did not reveal changes in the percentage of cycling thymocytes in cKO mice as compared to controls (4.5±0.4 vs. 4.2±0.7). In addition, no significant difference was found in the percentage of cycling cells among the four different thymocyte populations (data not shown). The sensitivity of the thymocytes to glucocorticoid-induced apoptosis was similar in cKO and control mice (data not shown). Analysis of spleen leukocytes did not reveal a significant change in the number of T cells (data not shown) or percentage of CD4^+^ and CD8^+^ T cells (Fig. 6B). The percentages of different T cell populations in cKO vs. controls were as follows. CD4^+^: 18±4.1 vs. 16±2.2 and CD8^+^: 9.5±4 vs. 7.1±2.9. There was also no significant change in the percentage of B cells (47.2±6.1 vs. 46.8±3.7).

### The intestinal barrier is not affected in cKO mice

**Figure 7:**
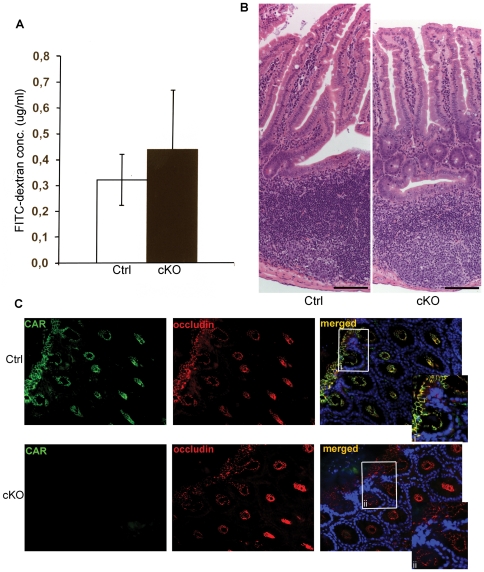
The integrity of the epithelial lining in intestines of cKO mice is maintained. (*A*) Tamoxifen-treated F/F;Cre (cKO, n = 11) and littermate F/F controls (Ctrl, n = 11) were given a fluorescent tracer by gavage and the permeability of the intestinal epithelium was analyzed by measuring the presence of fluorescence tracer in serum 4 hrs later. No statistically significant difference was found between the two groups. Data is shown as mean ± SD. *P* = 0.274. (*B*) Histology of small intestine harvested two weeks after the last tamoxifen administration from F/F control (Ctrl) and F/F;Cre (cKO) animals. No difference between cKO and control animals was found. Scale bar = 100 µm (*C*) Indirect immunofluorescence of large intestine from F/F;Cre (cKO) and littermate F/F control (Ctrl) mice two weeks after the last tamoxifen administration. Primary antibodies were directed against CAR (green) and occludin (red). DAPI was used to visualize nuclei (blue). Magnification was 40×. (i) and (ii) are enlargements of the boxed areas in the merged ctrl and cKO images, respectively. The experiment was performed three times.

The CAR protein is found in the tight junctions of most epithelial cells *in vivo* and *in vitro*. A decrease in the amount of CAR protein lowers transepithelial resistance and promotes diffusion of fluorescence markers across epithelial cell layers *in vitro* [4], [26]. To analyze if inactivation of the *Car* gene affects epithelial integrity also *in vivo*, the intestinal epithelium of tamoxifen-treated F/F;Cre (cKO, n = 11) and F/F control mice (Ctrl, n = 11) were challenged with fluorescent tracers administered by gavage. 4 hrs later, blood samples were taken and analyzed for the presence of fluorescent tracer. No statistically significant difference between cKO and control mice was observed indicating a maintained integrity of the intestinal epithelium (Fig. 7A). Similar results were obtained in animals devoid of CAR for up to 18 months (length of experiment). In addition, the cKO mice did not show signs of dehydration or oedema indicating that the skin barrier as well as the vascular system was intact.

Haematoxylin and eosin staining of the intestine did not reveal any differences between tamoxifen-treated control and cKO animals, and villi, crypts, Peyers patches, submucosa, muscle and epithelial layers in the cKO mice were all normal (Fig. 7B).

To analyze the tight junction protein complex at the molecular level, indirect immunofluorescence analysis was performed on intestinal epithelium using specific antibodies towards CAR and the junctional marker Occludin (Fig. 7C). Despite an almost complete lack of CAR in the epithelium of cKO mice, no difference was found between tamoxifen-treated cKO and control animals in Occludin expression. The same result was obtained when ZO-1, another tight junction associated protein, was analyzed (data not shown). These results indicate that the molecular composition of the tight junction complexes was unaffected in cKO mice.

Finally, we used transmission electron microscopy to analyze junctional complexes in epithelial tissue from the upper part of the intestine in tamoxifen-treated F/F;Cre (cKO, n = 3) and F/F control mice (Ctrl, n = 3). Although minor changes were occasionally found in cKO mice, the architecture of the junctions was in general maintained (data not shown).

Together these experiments indicate that epithelial integrity is maintained in CAR-deficient animals.

## Discussion

In this study we have created and analyzed a conditional *Car* knockout mouse, in which we have used the Cre-loxP system to disrupt the *Car* gene in adult mice. Previously reported *Car* knockout models have either targeted the *Car* gene in the germline causing an embryonic lethal phenotype, or used a cardiac-specific disruption of the CAR gene causing an embryonic lethal phenotype if induced during embryo development or a severe atrioventricular (AV) block if induced in adult mice [8], [9], [10], [11], [12]. This is the first study in which *Car* is ablated in all tissues in the adult mouse, enabling a more integrated analysis of the physiological role of the CAR protein *in vivo*.

Our results demonstrate that the *Car* gene can be irreversibly inactivated in a broad range of tissues in adult F/F;Cre mice in the presence of tamoxifen. One important exception is the liver, which maintained CAR expression also after repeated periods of tamoxifen administrations. The lack of tamoxifen-mediated Cre recombination in the liver has been described previously, but the reasons remain unknown [33].

A panel of experiments were performed to analyze the effect of CAR depletion on the major organ systems in adult mice. Several interesting phenotypes were observed, all irreversible with no difference between male and females (data not shown). No changes were observed in F/+;Cre heterozygotes (data not shown).

CAR-depleted mice demonstrated a striking atrophy of the exocrine part of the pancreas. Considering the known functional overcapacity of exocrine pancreas, it seems likely that the small areas of remaining unaffected parenchyma found in cKO mice was enough to maintain growth and longevity in cKO mice. In the cKO animals, numerous duct-like tubular complexes were observed. No tubular complexes were found in the remnants of normal exocrine pancreas in the cKO animals, indicating that acinar cell depletion is a prerequisite for the development of these complexes. The formation of tubular complexes//acinar metaplasia in the pancreas is thought to be a response to pancreatic injury and has been observed in several conditions including pancreatitis, chemically induced carcinogenesis, duct ligation, pancreatectomy, chronic hyperglycemia and animal models of diabetes [38], [39], [40], [41], [42], [43], [44], [45]. Although a transient infiltration of inflammatory cells was seen in the pancreas early after tamoxifen administration, this condition was not sustained indicating that the formation of tubular complexes in the cKO mice was not the result of chronic pancreatitis. In addition, no obstruction in ducts from the pancreas, liver or gall bladder and no malignant growth were observed in the cKO mice. Formation of tubular complexes in the pancreas has been suggested to originate from either proliferating pre-existing pluripotent cells, collapsed pre-existing ducts or from an acinar-to-ductal transdifferentiation process [46]. It was recently shown that exocrine cells might also transdifferentiate into ß cells via a tubular complex intermediate [47], [48]. Thus, tubular complexes appear to be unique structures harboring progenitor cells that, under appropriate conditions, might reconstitute islet ß-cells as well as exocrine pancreas as part of a tissue regenerative process.

It was recently demonstrated that mice with a pancreas-specific loss of *Car* from embryonic day 8 appeared normal [49]. We have created a similar mouse strain in which Cre expression is driven by the same pancreas-specific promoter [50]. In agreement with the results from Kallewaard et al, we could not demonstrate any abnormalities in the pancreas of these knockout mice in either the absence or presence of tamoxifen (data not shown). These results indicate that compensatory mechanisms must be operating in the pancreas if *Car* is deleted during embryo development. Alternatively, the pancreas-specific promoter may not express Cre in gall duct or other cells that might be important for CAR functions.

In addition to the atrophy of pancreas, cKO animals also displayed dilated intestines. The diameter along the whole intestinal system was significantly increased, while the length of the intestinal tract was normal. No signs of diarrhea, constipation, polyps, neoplasias or inflammation were observed. Histological analysis revealed a normal sized epithelium with no increase in cell proliferation in the crypts. The integrity of the intestinal epithelium was maintained in cKO animals. Together these results indicate that the enlarged intestines are not due to the lack of CAR in epithelial cells in the intestines, but instead might result from other alterations e.g. malfunctioning exocrine pancreas. Reduced secretion of pancreatic enzymes may result in an increase in remaining undigested material in the intestines and an increase in water content due to compensatory osmotic reactions. It is also possible that an altered motility of the gut affects the propulsion of the intestinal content. One mechanism could be an altered electrical activity of the smooth muscle or altered neuro/hormonal regulation of the gut motility. In view of the effects of CAR on impulse propagation in the heart, it could be possible that gap-junctional function in the gut is altered.

*Car* cKO mice showed a small, but statistical significant, increase in the total number of thymocytes in the thymus. No difference between cKO and controls animals in the rate of proliferation of any of the four main populations of thymocytes or in the sensitivity to glucocorticoid-induced apoptosis was found. The significance of this finding is at present unclear.

In agreement with previous reports, we show that disruption of the *Car* gene results in complete (or 3^rd^ degree) AV block with a full dissociation between atrial and ventricular excitation. The pacemaker responsible for the ventricular excitation is not identified. However, the ventricular rate and the shape of the QRS complexes are similar to those of the controls, which suggest that the pacemaker is located comparatively high in the conduction system. The faster ventricular frequency in cKO compared to control mice is in contrast to the bradycardia observed in mice following radiofrequency ablation of the AV node in experimental models of AV block, and in human AV block [51]. Our analysis included animals of different ages that had received different tamoxifen protocols. In all of these subgroups AV block was consistently observed in cKO mice, showing that conditional *Car* ablation results in a persistent loss of conduction in the AV node. No other signs of conduction failure (e.g. broadening of the qrs complexes) could be demonstrated. Histologically the heart appeared normal with no signs of increased proliferation, apoptosis, thoracic hemorrhage or late onset myocardial fibrosis. Transmission electron microscopy however revealed morphological changes with less well-defined contacts between myocytes in the cKOs. These results are well in line with the suggested role of CAR in organization of cell-cell contacts and the crosstalk between CAR and connexin proteins in the heart [12], [13].

The mechanisms behind the novel phenotypes reported in this study in not known. CAR is expressed in the intercalated discs of the heart, in tight junctions of the intestinal epithelium and the epithelium of pancreatic ducts, and in the epithelial reticular cells of the thymus [4], [11]. The localization of CAR to cell-cell contacts indicates that CAR might be important for the structural integrity of cells and tissues. Loss of CAR in the heart results in structural defects between myocytes and dysfunctional propagation of electrical signals. Cross talk between tight junctions and gap junctions is affected and mislocalization of connexins, ß-catenin and ZO-1 is observed [11], [12]. Our analyses of the intestinal epithelium did however not reveal any structural defects, leaking epithelium or mislocalization of Occludin or ZO-1 in the cKO mice. In addition, although leakage of the ductal epithelium in the pancreas was not directly analyzed, this scenario is less likely since a leakage of potent digestive enzymes into the surrounding tissue would probably cause pancreatitis, which was not observed in the cKO mice. Thus CAR might have function(s) beyond structural integrity. To reveal these function(s) requires further experimentation.

The multi-organ manifestation of the phenotype in *Car* cKO mice might reflect a syndrome associated with lack of function in the CAR protein. It is clear that one of the main functions of the protein is related to tight junctional activity as manifested by the clear cardiac phenotype. Our novel finding that the pancreas is dramatically affected and that the intestine is dilated suggests that a human equivalent phenotype would exhibit clinical symptoms from both heart and the gastro-intestinal system. Obviously, global deletion of CAR affects embryonic development and a global ablation might not be relevant for conditions in man. Since human pathological genotypes often are heterozygous, milder symptoms in man might be expected.

In conclusion, this paper confirms the previously reported function for CAR in conduction of electric signals in the heart. We also report novel functions of CAR in maintaining the exocrine pancreas and possible also intestines and thymus of adult mice. All phenotypes show a 100% penetrance and appear to be irreversible. Future studies are needed to address the mechanism(s) behind these phenotypes and link the mouse phenotypes to a clinical genetic syndrome or other, possibly virus related, changes in CAR in man.
